# Reply to: Perceptions of intensive care unit health care professionals in Brazil regarding postintensive care syndrome: a survey study

**DOI:** 10.62675/2965-2774.20260135

**Published:** 2026-07-21

**Authors:** José Mário Meira Telles, Cassiano Teixeira, Regis Goulart Rosa

**Affiliations:** 1 Hospital Municipal de Salvador Salvador BA Brazil Hospital Municipal de Salvador – Salvador (BA), Brazil.; 2 Universidade Federal de Ciências da Saúde de Porto Alegre Internal Medicine and Rehabilitation Sciences Department Porto Alegre RS Brazil Internal Medicine and Rehabilitation Sciences Department, Universidade Federal de Ciências da Saúde de Porto Alegre - Porto Alegre (RS), Brazil.; 3 Universidade Federal do Rio Grande do Sul Intensive Care Department Porto Alegre RS Brazil Intensive Care Department, Universidade Federal do Rio Grande do Sul - Porto Alegre (RS), Brazil.

**DEAR EDITOR,**

We thank Nav La et al.^(1)^ for their thoughtful and constructive comments on our study, "Perceptions of intensive care unit health care professionals in Brazil regarding postintensive care syndrome: a survey study."^(2)^ Yours observations add an important organizational lens to our findings and help to broaden the discussion about the challenges of postintensive care unit survivorship care in Brazil.

We welcome the opportunity to emphasize key concordant points. Our data consistently show that while most intensive care unit (ICU) professionals recognize the long-term consequences of critical illness, institutional structures to address these needs are often lacking: only 26.4% of respondents reported formal institutional protocols for assessing post-discharge needs; ICU teams were not involved in the discharge process in 60% of cases; and systematic pre-discharge screening for high-risk patients and family members was reported by only 14.7% and 8.7% of respondents, respectively. These findings underscore a clear mismatch between individual awareness and organizational implementation, and they support the view that recognition alone is insufficient without structured institutional pathways for screening, transition planning, and follow-up.

We appreciate the perception-action gap framework you propose and find it useful. Although our cross-sectional survey was not designed to test causal pathways formally, the framework offers a helpful conceptual lens: high individual prioritization of patient- and family-centered outcomes contrasted with perceived lower institutional prioritization - differences that were statistically significant in our analyses - suggests misalignment that warrants targeted organizational strategies. We also welcome the emphasis you place on the postintensive care syndrome-family; our results indicate that family-centered outcomes are consistently valued by clinicians but are perceived as less prioritized by institutions, highlighting a practical area for intervention.

We are grateful for the opportunity to clarify several methodological limitations of our work and to suggest how future studies might address them. First, our recruitment strategy (beginning at a national critical care congress and extending through *Associação de Medicina Intensiva Brasileira* [AMIB] channels and social media) relied on convenience sampling, which may overrepresent more engaged professionals. Future research would benefit from probabilistic or stratified sampling across regions, institution types, and professional categories, using administrative lists in which possible and reporting response rates. Second, physicians comprised 51.1% of respondents, potentially limiting the representativeness of nurses, physiotherapists, psychologists, and social workers who play crucial roles in prevention and rehabilitation; quota sampling, targeted recruitment of underrepresented professions, and professionstratified analyses (or weighted adjustments) could mitigate this. Third, the cross-sectional design limits causal inference; longitudinal cohort studies, before-and-after evaluations of institutional interventions, or mixed-methods designs could better elucidate causal pathways and mechanisms. Fourth, dichotomizing Likert responses may have reduced nuance and statistical power; preserving ordinal data and applying ordinal regression or sensitivity analyses comparing categorical *versus* dichotomized approaches would retain granularity. Fifth, instruments adapted for local use should undergo formal psychometric validation (pilot testing, internal consistency, construct validity, factor analysis) to ensure contextual relevance. Sixth, reliance on selfreport may introduce information bias regarding institutional practices; triangulation with institutional records, protocol audits, or complementary qualitative interviews would strengthen validity. Finally, given Brazil's regional and public/private heterogeneity, stratified sampling by geography, hospital size, and funding source, and pre-specified subgroup analyses would improve generalizability.

In closing, we appreciate your engagement with our work and the thoughtful suggestions you have offered. We agree that future efforts should move beyond awareness to develop and evaluate structured discharge processes, multidisciplinary transition planning, routine family assessment, and sustainable post-ICU follow-up models - especially tailored to middle-income health systems. We look forward to integrating these considerations into subsequent research and to continued dialogue on effective strategies to improve survivorship care.
